# Alleviating the toxic effects of Cd and Co on the seed germination and seedling biochemistry of wheat (*Triticum aestivum* L.) using *Azolla pinnata*

**DOI:** 10.1007/s11356-023-27566-1

**Published:** 2023-05-26

**Authors:** Zeinab A. Shedeed, Emad A. Farahat

**Affiliations:** Botany and Microbiology Department, Faculty of Science, Helwan University, Cairo, 11795 Egypt

**Keywords:** Heavy metals, Wheat, *Azolla pinnata*, Water pollution, Cd and Co pollution, Enzymes

## Abstract

One of the most significant environmental challenges in the twenty-first century is heavy metal pollution. The potential use of fresh *Azolla pinnata* to alleviate the toxic effects of Cd and Co on the germination measurements of wheat seeds (*Triticum aestivum* L.) and the biochemistry of seedlings was studied. Two concentrations (80 and 100 mg L^−1^ solutions) of CdNO_3_ and CoCl_2_ were used before and after treatment with *A. pinnata*. The highest removal efficiency (RE) by *A. pinnata* was obtained on the fifth day, with a Cd RE = 55.9 and 49.9% at 80 and 100 mg L^−1^, respectively. Cadmium and cobalt solutions reduced the germination percentage, and the measured variables of wheat seeds meanwhile increased the radicle phytotoxicity. In contrast, the presence of *A. pinnata* in the germination medium increased all the measured variables and decreased radicle phytotoxicity. At 80 and 100 mg L^−1^, Cd significantly reduced the fresh and dry biomass, and height of wheat seedlings after 21 days of cultivation compared to Co. Cadmium and high concentrations of cobalt increased the contents of H_2_O_2_, proline, MDA, phenolic, and flavonoid compounds. The application of treated Cd and Co solutions by *A. pinnata* showed a decrease in H_2_O_2_, proline, phenolic, and flavonoid compounds levels accompanied by a reduction in catalase and peroxidase activities compared to the control. This study showed the positive role of *A. pinnata* in alleviating the metal impacts, particularly Cd, on the seedling growth of wheat and its germination.

**Supplementary Information:**

The online version contains supplementary material available at 10.1007/s11356-023-27566-1.

## Introduction

The presence of organic and inorganic contaminants leads to many hazards and negative consequences in the environment, which has become a serious problem that threatens the global ecology (Zulfiqar et al. 2019; Zeeshan et al. 2021). The accumulation of heavy metals in soil is one of the primary causes of pedosphere degradation. Heavy metals are found in nature as a non-degradable part within the Earth’s crust (Mahey et al. 2020). Heavy metals/metalloids (e.g., Cd and Co) have resulted from either natural or man-made sources such as byproducts from the oil and gas sectors (Pichtel 2016), and agricultural use of phosphate fertilizers (Kubier et al. 2019). The small amounts of heavy metals in the soil are available to plants as soluble components (Blaylock and Huang 2000). Heavy metals/metalloids may be categorized into two types based on their bioavailability in soil: highly bioavailable heavy metals (Cd, Ni, Zn, As, Se, Cu) and moderately bioavailable heavy metals (Co, Mn, Fe) (Yan et al. 2020).

There are three main routes for Cd_2_C entrance into the root; the first is the plasma membrane of root epidermal cells, where absorbed H^**+**^ exchanges fast with Cd_2_C, and adsorbed on the surface of root epidermal cells. Second, Cd_2_C enters plant cells via the same ion channels that allow Fe_2_C, Zn_2_C, and Ca_2_C. Third, plant roots actively produce mugineic acid (Cd_2_C chelator), which chelates Cd_2_C to increase ion availability in the soil rhizosphere. Cd_2_C reaches the root epidermal layer as chelates via yellow-stripe 1-like (YSL) proteins (Song et al. 2017). In addition, Co-specific transporters have not been reported in plants. Co is either sequestered in the vacuole of root cells or transported to shoots after absorption by roots. The transported Co to the shoots is chelated with ligands (Hu et al. 2021).

Cadmium and other heavy metals (HMs) cause oxidative damage in plants by creating excessive H_2_O_2_ and causing lipid peroxidation (Shiyu et al. 2020; Yang et al. 2020). Redox-active metals such as Co, Cr, Mn, Fe, and Cu are those that can cause cell damage by directly creating reactive oxygen species (ROS) via the Haber–Weiss and Fenton reactions (Valko et al. 2005; Ozefczak et al. 2012). On the other hand, metals such as Cd, Ni, Hg, Zn, and Al can harm cells and their subcellular vitals by indirectly increasing ROS levels via glutathione depletion. This leads to blocking antioxidant enzymes, binding active sulfhydryl groups of proteins, and activating ROS-generating enzymes such as NADPH oxidase (Bielen et al. 2013).

Metal stress tolerance is strongly associated with ROS detoxification enzymes and components such as metal chelators (Gratão et al. 2019). There is a defense system including ROS scavenging enzymes (enzymatic antioxidants) and compounds (non-enzymatic antioxidants) responsible for protecting the cells during the extensive production of ROS (Rajput et al. 2021). For example, proline content increased in plants that are exposed to high metal concentration (Sharma and Dietz 2006). In vitro, proline can get rid of ^1^O_2_ and ^•^OH radicals, and in vivo studies have shown that it also acts as an antioxidant when there is metal stress (Signorelli et al. 2013).

The addition of *Azolla* into agriculture soil before or after transplanting was recommended by Kimani et al. (2020) to improve soil fertility and reduce the demand for synthetic fertilizer. Contrasting other biofertilizers, *Azolla* has been shown to recover a wide range of macronutrients and micronutrients in paddy fields, including Ca^2+^, Mg^2+^, P, Fe, S, and K^+^ (Nayak et al. 2004). *Azolla* biofertilizer extract is also utilized as a foliar fertilizer in crops like tomatoes (Hanafy et al. 2018), as well as cereals like maize (*Zea mays*) (Maswada et al. 2021) and roselle (*Hibiscus sabdariffa*) (Al-Sayed et al. 2022). One of the *Azolla*’s favorable benefits is that it enriches soil organic matter, improves soil quality, and supplies fixed nitrogen. Additionally, humus is formed due to the decomposition of *Azolla*, which enhances the soil’s water-holding capacity and promotes aeration and drainage (Bhuvaneshwari and Kumar 2013). Unfortunately, global *Azolla* use is still undesirable in social, economic, and scientific backing, leading farmers to lose faith in the technique and rely more on artificial fertilizers.

To prevent the pollution of heavy metals through the food chain, it is critical to remediate or alleviate the contaminated water with heavy metals that are used in the irrigation of edible crops. *Azolla pinnata* as water ferns are known for their potential use in the phytoremediation of heavy metals from contaminated water (Naghipour et al. 2018; Kumar et al. 2020). Therefore, the objective of this study was to investigate the potential use of fresh biomass of *A. pinnata* as a phytoremediator to Cd or Co in the growing medium or irrigation water of wheat seeds and seedlings, respectively. We hypothesize that *A. pinnata* can help wheat to germinate and grow normally under Cd or Co water pollution.

## Materials and methods

### Sterilization of seeds and preparation of solutions

Seeds of wheat (*Triticum aestivum* L.) and *A. pinnata* were kindly provided by the Agriculture Research Center, Giza, Egypt, after proper identification by the scientific staff. Wheat seeds were surface sterilized for 5 min in a 2% solution of sodium hypochlorite. Then, they were thoroughly washed several times with distilled water to remove all traces of the disinfecting solution (Bajji et al. 2011). Uniform seeds were selected for the germination and pot experiment. Cd and Co solutions of two concentrations (80 and 100 mg L^−1^) were prepared with distilled water using cadmium nitrate (CdNO_3_) and cobalt chloride (CoCl_2_), respectively. These solutions were used in the irrigation of wheat seeds and seedlings during the experiment as will be mentioned below.

### Removal efficiency assay of Cd and Co from their solutions by A. pinnata

The fresh *A. pinnata* was washed first with distilled water and sterilized with Mercuric chloride (0.1%) for 30 s, then washed with distilled water 7–10 times. Then, the treated *A. pinnata* was plotted between two sheets of tissue paper and air-dried for about 15 min. Twenty grams of *A. pinnata* was added to 200 ml of each Cd (II) and Co (II) solution (80 and 100 mg L^−1^) in 250 mL beakers. Then, all beakers were incubated at 25 °C for 5 days. For the following five days, 10 mL samples from each treatment were taken and preserved in a capped tube at − 20 °C for analysis. All the taken samples were analyzed for Cd or Co by microwave plasma atomic emission spectrometer (MP-AES, Agilent, USA) at Ecology laboratory, Helwan University according to the manufacturer’s guidelines.

### Germination experiment

The selected uniform wheat seeds were put on 9 cm diameter filter paper in sterile Petri dishes with water (control) or the assigned concentrations of Cd and Co (80 and 100 mg L^−1^). For each treatment, three replicates (Petri dishes) of 15 seeds were used. The fresh biomass (10%) of *A. pinnata* (i.e., 10 g/100 mL metal solution) was added to the Petri dishes used in the germination experiment, beneath the filter paper and before adding the different concatenations of Cd and Co solutions. The seeds were incubated at 25 °C and irrigated daily with 3 mL of the appropriate Cd or Co solution or distilled water (control). The germination percentage, radicle and coleoptile length, α-amylase activity, and total soluble sugar content were determined on the fourth day of germination.

### Pot experiment

Seeds (5 seeds) of wheat were sown in separate plastic pots (30 cm diameter). Each pot was filled with 1 kg of Patmos. The chemical structure of Patmos is provided as supplementary Table S1. The pots were divided into five groups that represent the treatments (control, raw Cd and Co solutions, and *Azolla*-treated Cd and Co solutions). Each group represents a treatment for the two concentrations of 80 and 100 mg L^−1^). The studied five treatments for control, Cd, and Co (80 and 100 mg L^−1^ each) were represented by 15 pots for each treatment (3 replicates, *N* = 5 pots). For control plants, water was applied during the whole period of the experiment. The prepared raw and *Azolla*-treated Cd and Co solutions in 80 and 100 mg L^−1^ concentrations were applied twice a week at a 60% field capacity to the pots after 1 week of cultivation. The irrigation schedule for each group was as follows:

1. First group (control): water was applied to pots during the whole period of the experiment.
2. Second group, untreated 80 and 100 mg L^−1^ of Cd solutions were applied twice a week after one week of cultivation (*N* = 3).
3. For the third group, *A. pinnata*-treated solutions (on the fifth day) 80 and 100 mg L^−1^ of Cd solutions were applied twice a week after 1 week of cultivation.
4. For the fourth group, the untreated 80 and 100 mg L^−1^ of Co solutions were applied after 1 week of cultivation.
5. For the fifth group, *A. pinnata*-treated solutions (on the fifth day) 80 and 100 mg L^−1^ of Co solutions were applied after one week of cultivation.

Two weeks after the application of the heavy metals’ solutions, the wheat plants from each pot were collected and used for measuring their fresh and dry biomasses, plant height, and the number of leaves/plant. Subsets of sampled fresh leaves for each treatment were used for enzyme activity bioassay, determination of H_2_O_2_, MDA (malondialdehyde), proline content, and total antioxidant capacity. Dry leaves powder was used in the determination of total phenolic and flavonoid compounds content.

### Biochemical analysis and assay of enzymes

Endogenous H_2_O_2_ content was determined according to the modified method of Velikova et al. (2000). Determination of proline content was conducted according to the method of Bates et al. (1973) by using a ninhydrin reagent. Lipid peroxidation was measured in the form of malondialdehyde (MDA) content, the product of lipid peroxidation according to Heath and Packer (1968). Total soluble sugar content was measured by using an anthrone reagent (2 g L^−1^ H_**2**_SO_**4**_) (Umbreit et al. 1959). Total phenolic content was determined with the Folin-Ciocalteu method (Singleton et al. 1999). Total flavonoid content was estimated by Zhishen et al. (1999) method. The total antioxidant capacity was determined according to Prieto et al. (1999).

To measure the amylase enzyme, about 4 g of plant material was homogenized with 20 ml of sodium phosphate buffer, pH 7.5. The homogenate was vortexed and centrifuged for 10 min at 20,000 g at 4 °C in a cooling centrifuge, and the supernatant was used for α-amylase assay (Sangeetha 2013). α-Amylase (E.C. 3.2.1.1) activity was determined by Rick and Stegbauer’s method (1974).

Catalase (E.C.1.11.1.6) was extracted and measured according to the method of Góth (1991). Peroxidase (E.C. 1.11. 1. x) was measured according to Chance and Maehly (1955) by the guaiacol oxidation method. Superoxide dismutase (E.C. 1.15.1.1) was assayed by the method described by Dhindsa et al (1981).

### Statistical analysis

The significance of growth and biochemical parameters at different treatments of wheat was evaluated using a one-way analysis of variance (ANOVA I). When the differences are significant, a post hoc test (Tukey test at *P* < 0.05) was applied using the SPSS software V.15.0 user’s guide (IBM SPSS Statistics).

## Results and discussion

**Fig. 1:**
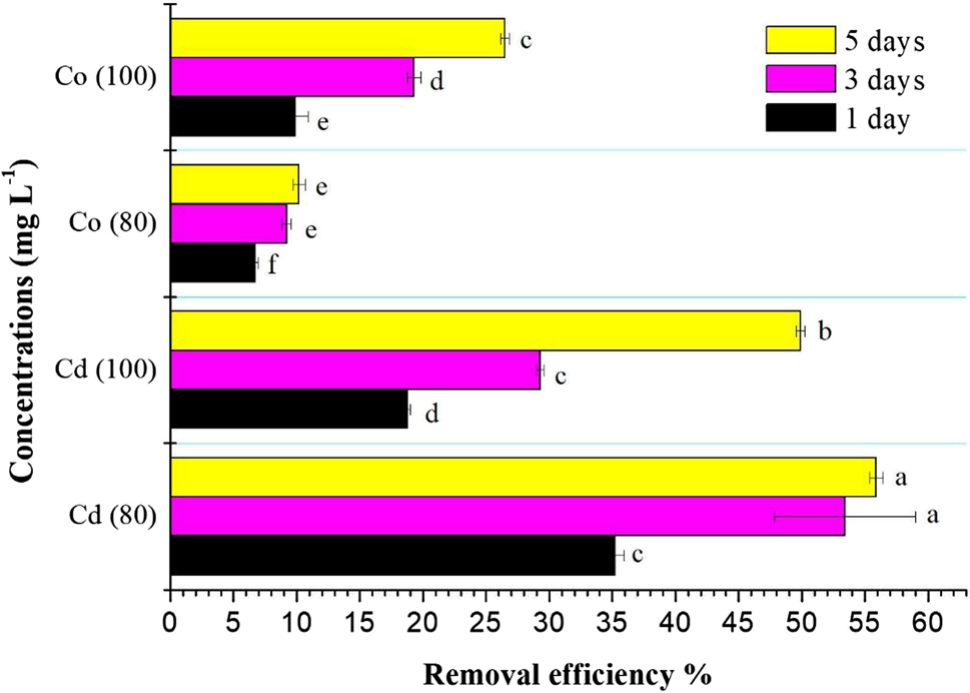
The removal efficiency of cadmium (Cd) and cobalt (Co) by *A. pinnata* (10%) from original solutions (80 and 100 mg L^−1^) at the first, third, and fifth days of incubation. Means with different letters for each treatment are significantly different at *P* < 0.05. Standard error bars are shown above bars

The exclusion of contaminants from agricultural ecosystems is a vital process to avoid their transfer to our food chain. Figure 1 shows the removal efficiency percentage (RE) of Cd and Co metals from their initial solutions of 80 and 100 mg L^−1^ by *A. pinnata*. The results showed a gradual increase in the RE % of metals (Cd and Co) from initial solutions by *A. pinnata* for different concentrations with the increase of the incubation period from one to five days. Similarly, Kumar et al. (2020) found that the removal percentage of Cd, Cu, Fe, Cr, and Zn by *A. pinnata* from 60% of integrated industrial effluent (IIE) was 57.27, 53.85, 56.06, 58.06, and 60.03%, respectively. Pandey (2012) found that the accumulation rates of heavy metals by *A. caroliniana* from a fly ash pond were as follow: Fe (343.7) > Mn (76.5) > Zn (45.1) > Cd (27.0) > Ni (21.8) > Cu (19.3) > Pb (16.2) > Cr (12.5).

As a starting stage in the plant life cycle, seed germination is a determinant stage for plant individuals, populations, and the achievement of crop productivity (Al Khateeb et al. 2010). The results in Table 1 showed that the presence of Cd and Co concentrations in the germinating medium (80 and 100 mg L^−1^) leads to a significant reduction in the germination percentage (maximum reduction = 36%), and radicle (maximum reduction = 76.9%) and coleoptile (maximum reduction = 94.6%) lengths (at *P* ˂ 0.05). In addition, α-amylase and total soluble sugar contents were negatively affected by different concentrations of Cd and Co. It was observed that Cd decreased remarkably the germination percentage, lengths of coleoptile and radicle, α-amylase activity, and soluble sugar content compared to Co. Rahoui et al. (2010) reported that Cd and Co delay germination, affecting food reserve mobilization by increasing total soluble sugar and amino acid ratios in cotyledons/embryos.

**Table 1:** Effect of untreated and treated Cd and Co — (80 and 100 mg L^−1^) with *A. pinnata* (10%) on germination percentage (%), radicle and coleoptile lengths (cm), radicle phytotoxicity (%), α-amylase activity (mg g^−1^ protein min^−1^), and total soluble sugar content (mg g^−1^ F wt) of wheat seedlings at the fourth day of germination. Means with different letters in each column are significantly different at *P* < 0.05

Element	Concentration, mg L^−1^	Treatment	Germination, %	Radicle L	Coleoptile L	Radicle Phytotoxicity (%)	α-amylase	Soluble sugar
0 (Control)			100 ± 0.001 a	7.8 ± 0.4 a	6.2 ± 0.26 a	0	15.50 ± 0.5 a	30.36 ± 0.64 a
Cd	80	Untreated	87.5 ± 0.45 d	2.8 ± 0.28 f	0.63 ± 0.05 e	63.5 ± 4.8 b	10.86 ± 0.23 e	25.25 ± 0.45 d
	100		64.0 ± 5.1 g	1.8 ± 0.20 f	0.33 ± 0.05 e	76.7 ± 2.9 a	5.66 ± 0.57 g	16.80 ± 0.45 f
	80	Treated	95.5 ± 0.40 b	5.6 ± 0.30 d	4.2 ± 0.25 b	27.7 ± 6.7 d	11.93 ± 0.11 d	30.03 ± 0.17 a
	100		81.2 ± 0.25 e	3.8 ± 0.23 e	3.0 ± 0.20 c	50.7 ± 3.7 c	9.83 ± 0.28 f	19.76 ± 0.25 e
Co	80	Untreated	91.8 ± 0.70 c	6.1 ± 0.34 cd	0.93 ± 0.05 d	26.2 ± 8.2 c	14.83 ± 0.28 b	30.63 ± 0.55 a
	100		75.1 ± 0.15 f	4.2 ± 0.28 e	0.53 ± 0.04 e	46.6 ± 6.0 de	11.00 ± 0.4 e	27.83 ± 0.15 c
	80	Treated	98.4 ± 0.47 ab	6.9 ± 0.15 b	4.50 ± 0.25 b	12.5 ± 4.0 f	15.53 ± 0.05 a	30.20 ± 0.69 a
	100		90.3 ± 0.11 cd	6.5 ± 0.05 bc	3.00 ± 0.2 c	17.6 ± 4.1 ef	13.93 ± 0.11 c	29.65 ± 0.28 b

Means with different letters in each column are significantly different at *P* < 0.05

Cadmium and cobalt stress causes a decrease in the activity of hydrolytic enzymes, which results in a decrease in starch release from the cotyledons to the embryo axis (Sanal et al. 2014). The decrease in water supply, inactivation of endosperm starch mobilization, and poor transport of soluble carbohydrates to the seed embryonic axis can all lead to further hunger on the embryonic axis (Kuriakose and Prasad 2008). In general, heavy metals decrease enzymatic hydrolysis (especially by α-amylase) of starchy endosperm, which stops the translocated simple sugar to developed embryo axes (Mittal et al. 2015). In the present study, the coleoptile was more sensitive to Cd and Co stress than the root. The shoot inhibition percentage was 94.6 and 91.4% for Cd and Co, respectively, at 100 mg L^−1^. Many plant species have shown the same result towards different heavy metal stresses (Ishtiaq and Mahmood 2011). The present study revealed that the phytotoxicity (Table 1) was significantly (*P* ˂ 0.05) increased by increasing the Cd or Co concentration from 80 to 100 mg L^−1^. Mondal et al. (2013) reported a decrease in the root development of chickpeas (*Cicer arietinum*) under Cd stress. The authors also mentioned that the decrease in stem length may be caused by the direct suppression of cellular elongation or division, slowed root development, and decreased transfer of nutrients and water.

Adding the fresh biomass of *A. pinnata* to the wheat growing medium had a positive effect during germination under Cd and Co stress (Table 1). There was an enhancement of germination percentage, coleoptile, and root lengths, α-amylase activity, and total soluble sugar content due to the presence of *A. pinnata* compared to the negative control. Besides, the addition of *A. pinnata* to the germination medium led to a reduction of radical phytotoxicity by > 50% compared to the untreated solution. Jayasundara (2022) reported that *A. pinnata* is a good phytoremediator for heavy metals from wastewater. The uptake of metals by aquatic plants such as *A. pinnata* depends on two mechanisms: firstly, the developed surface reaction via diffusion process by the fast metabolic rate led to finally the soluble metal-binding or adsorbing to the cell wall of the aquatic plant. Secondly, the developed mass transport depends on cellular uptake during a slower metabolism from the outer cell wall to the cell (Solanki and Dhankhar 2011). The charged groups as the carboxyl group on the cell wall are the site of the primary bind and replacement of the ions of the heavy metal to the non-soluble components in the *A. pinnata* cell wall components. For instance, pectin and cellulose interact with Mg and Ca, which are considered the exchanger ions with the heavy metal forming a three-dimensional polymer (Kamnev et al. 1998).

**Fig. 2:**
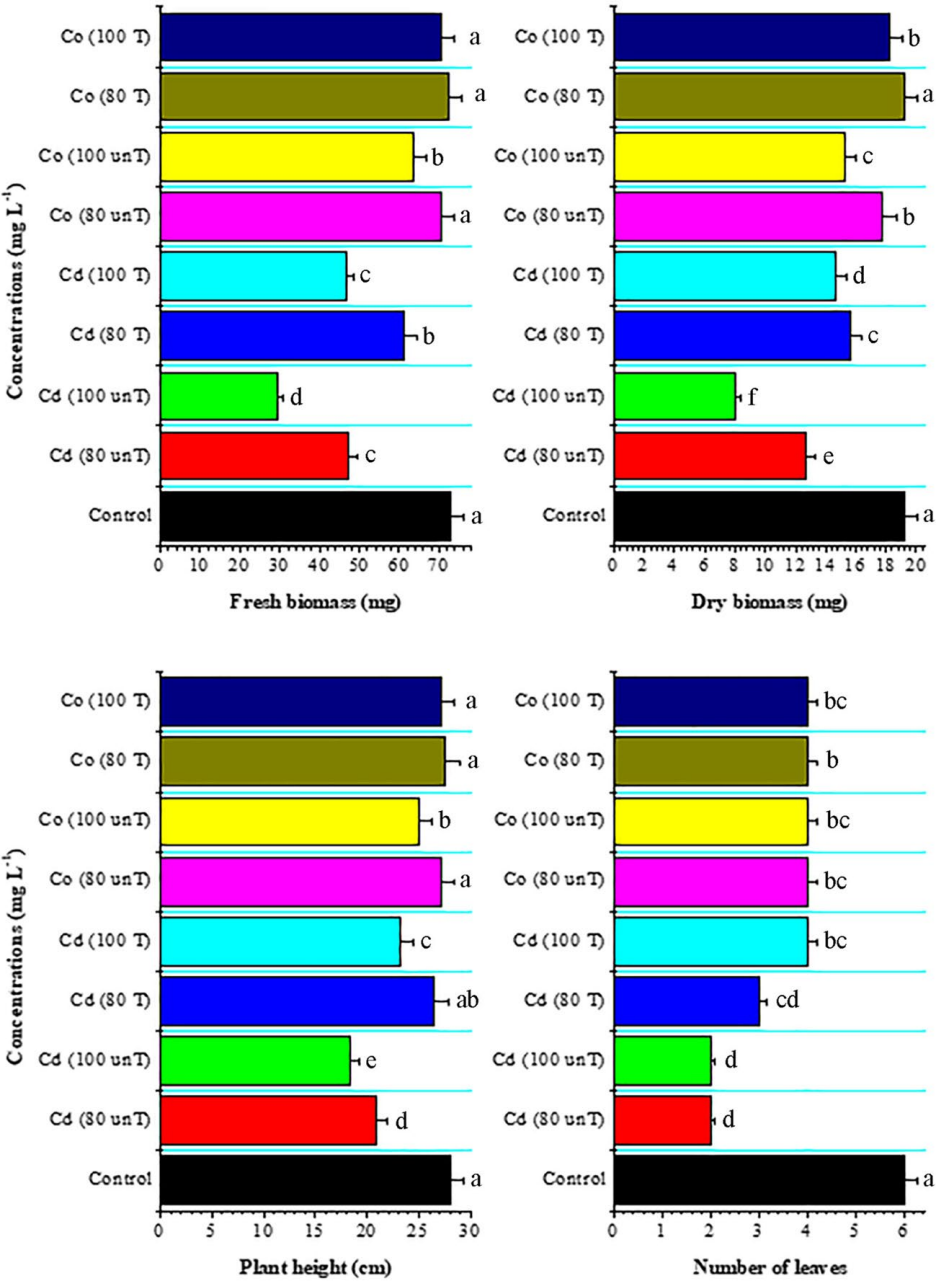
Effect of untreated (unT.) and treated (T) Cd and Co solutions (80 and 100 mg L^−1^) with *A. pinnata* (10%) on growth parameters of wheat seedlings after 21 days of cultivation. Means with different letters for each treatment are significantly different at *P* < 0.05. Standard error bars are shown above bars

The application of Cd and Co contaminated solutions on wheat plants had an obvious effect on their growth parameters as shown in Fig. 2. Cd treatment negatively affected the fresh and dry biomasses, plant height, and the number of plant leaves at 80 and 100 mg L^−1^. In contrast, Co-treatment had no significant effect on the fresh biomass or plant height at 80 mg L^−1^. Co-treatment had a more inhibitory effect on growth parameters at 100 mg L^−1^ concentration compared to other treatments. Zulfiqar et al. (2022) reported that Cd toxicity harms plants in a variety of ways depending on time and concentration. Cd damages membranes and interferes with photosynthetic and enzymatic activities. Moreover, Cd causes oxidative damage to cells and induces structural changes in the photosynthetic apparatus, which negatively affects the final yield. In contrast, it has been proposed that Co supports several developmental processes, including stem and coleoptile elongation, hypocotyl hook opening, and leaf disc expansion and growth (Vaseer et al. 2019). However, excessive dosage of Co may negatively affect plant development (Hu et al. 2021). This can explain the high growth inhibitory effect of Co on wheat seedlings at 100 mg L^−1^ in the present study.

Application of treated Cd and Co solutions by *A. pinnata* on wheat ameliorates the growth parameters of the plants as shown in Fig. 2. High values were obtained for fresh and dry biomasses and plant height of wheat by the application of treated Cd and Co solutions compared to the untreated ones. Similarly, Rai and Tripathi (2008) suggested the use of *A. pinnata* to remediate contaminated water by Cd (II). In the present study, the high Co concentration of 100 mg L^−1^ caused a significant reduction in the growth parameters of wheat plants compared to 80 mg L^−1^ concentration (Fig. 2). Accordingly, the application of *A. pinnata* was more effective at 100 mg L^−1^ of Co solutions. By the application of treated solutions on wheat plants, the fresh and dry biomass and height of plants were increased and close to the control values.

**Fig. 3:**
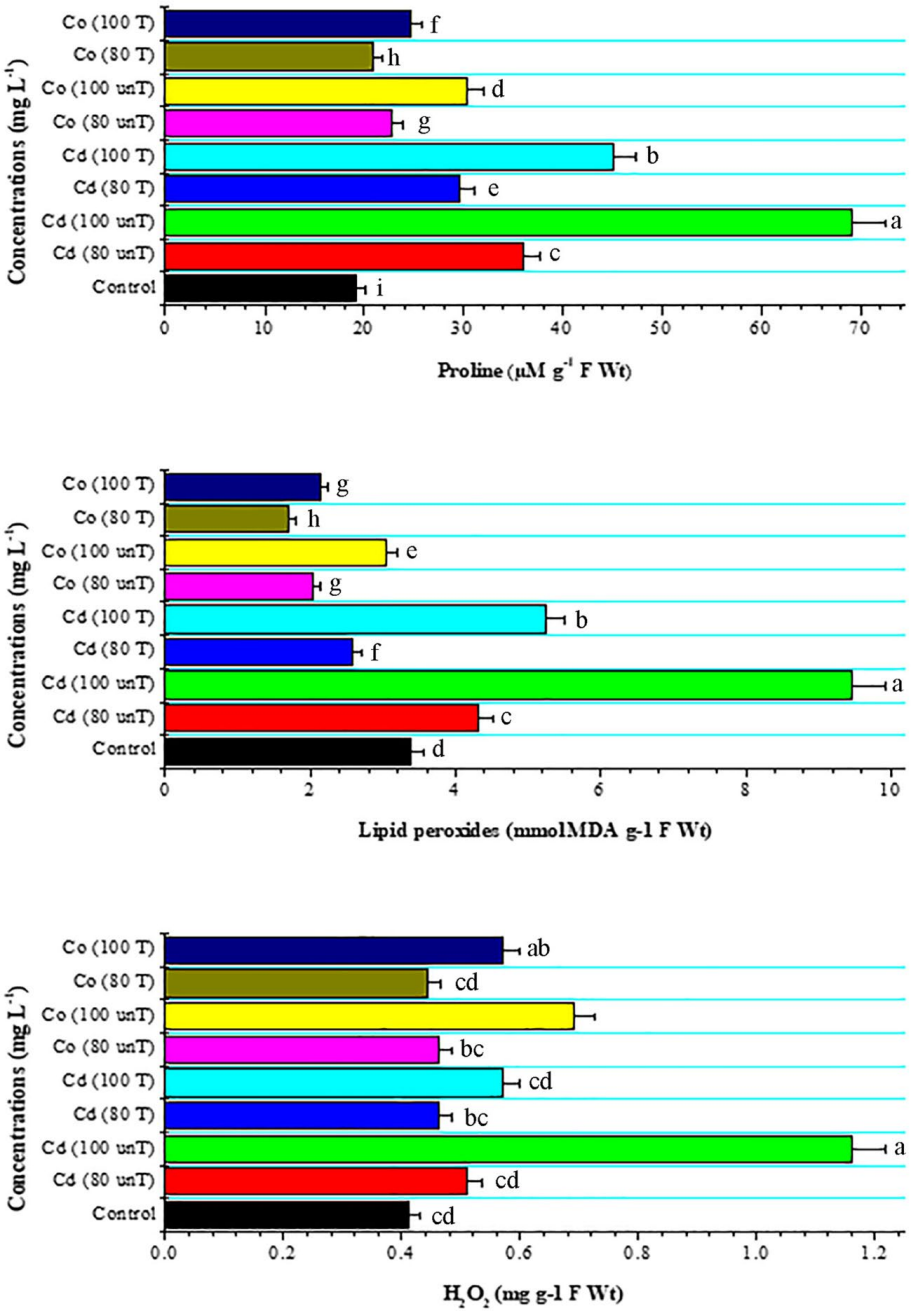
Effect of untreated (unT.) and treated (T) Cd and Co solutions (80 and 100 mg L^−1^) with *A. pinnata* (10%) on proline (µM g^−1^ F wt), lipid peroxides (mmol MDA g^−1^ F wt), and H_2_O_2_ (mg g^−1^ F wt) content of wheat seedlings after 21 days of cultivation. Means with different letters for each treatment are significantly different at *P* < 0.05. Standard error bars are shown above bars

Because reducing the heavy metal content of water is difficult, only oxidation state transformation is applicable (Singh et al. 2018). Eid et al. (2020) reported that *A. pinnata* can be used as an eco-friendly phytoremediator for heavy metals from water and soil. In response to biotic and abiotic stress, the high production of ROS such as H_2_O_2_, MDA, and proline is stimulated in plant cells and released in the apoplast (Sahu et al. 2022). The present study revealed that increasing Cd and Co concentrations leads to the accumulation of H_2_O_2_, MDA, and proline content in wheat seedlings to reach their high levels at 100 mg L^−1^ (Fig. 3). Anjum et al. (2015) and Khan et al. (2007) found that Cd stress produces hydrogen peroxides (H_2_O_2_), hydroxyl radicals (^●^OH), and superoxide anion (^●^O_2_), all of which cause membrane damage. Under Cd stress, the formation of thiobarbituric acid reactive substances (TBARSs) and malondialdehyde (MDA) develops as a consequence of oxidative damage, resulting in electrolyte leakage (Younis et al. 2016). Moreover, Co stress increases membrane permeability, H_2_O_2,_ and MDA concentration, and proline buildup (Samet 2020). As an excellent osmolyte, proline performs crucial roles under stress as a metal chelator, a molecule of antioxidant defense, and a molecule of signaling (Hosseinifard et al. 2022). To adapt to stress in plants, proline performs a variety of roles, including adaptation, recovery, and signaling (Kaur and Asthir 2015).

**Fig. 4:**
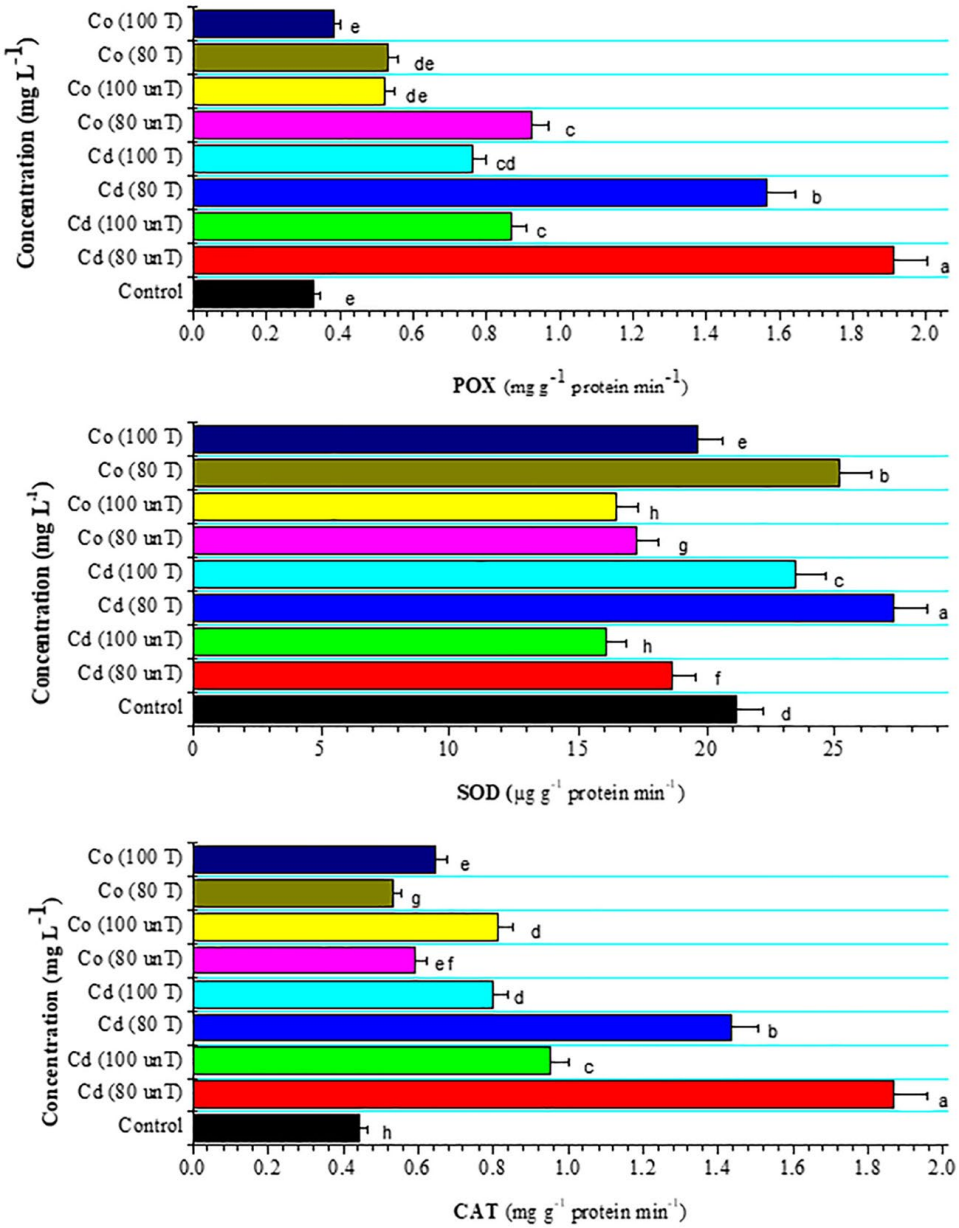
Effect of untreated and treated Cd and Co solutions (80 and 100 mg L^−1^) with *A. pinnata* (10%) on peroxidase (POX, mg g^−1^ protein min^−1^), catalase (CAT, mg g^−1^ protein min^−1^), and superoxide dismutase (SOD, µg g^−1^ protein min^−1^) activities of wheat seedlings after 21 days of cultivation. Means with different letters in each column are significantly different at *P* < 0.05. Standard error bars are shown above bars

In the present study, the activity of the antioxidant enzymes peroxidase (POX) and catalase (CAT) activity was gradually increased by elevating Cd and Co levels in the growth medium (Fig. 4). In Co-toxic levels to plants, the enzymatic activity of CAT increased. One probable explanation for this result is to minimize oxidative damage. A significant increase in cell membrane damage might be linked to a significant imbalance in the activity of antioxidative enzymes for ROS scavenging. In Girish et al. (2009) study, they found that the efficiency in producing lipid peroxidation was in the following order: Ni > Co > Cd > Cu > Zn, in line with the degree of observable toxicity effects and reduction in dry matter yield. The CAT and POX enzymes are responsible for the conversion of H_2_O_2_ to water and oxygen via H_2_O_2_ dissociation, and hence, they play important roles in plant tolerance to adverse environments (Li et al. 2014). Hassan et al. (2020) demonstrated that sorghum cultivar JS-2002 maintained greater POX and CAT activities than Chakwal sorghum during Cd toxicity (25 M), indicating that JS-2002 has a stronger antioxidant ability. A significant rise in POX and CAT activities might be triggered by the high H_2_O_2_ generation. On the other hand, Jaleel et al. (2008) reported an inhibition and stimulation of the CAT and POX activities, respectively, in *Arachis hypogaea* seedlings under Co stress.

The results showed that the superoxide dismutase (SOD) activity decreased at a low metal concentration (80 mg L^−1^) and increased at a high metal concentration (100 mg L^−1^) treatment of Cd and Co (Fig. 4). The increased synthesis of O_2_ radicals, which leads to the activation of existing enzyme stock, might be one of the causes of the elevation as reported by Zhang et al. (2009). In addition, SOD activity was parallel to ROS level to avoid their accumulation under stress. This finding was in line with Hassan et al. (2020), who reported a decline of SOD at 5, 25, 50, and 100 mM of Cd concentrations followed by an increment of its activity in sorghum seedlings. Dey (2008) found that the elevation of Cd led to a remarkable reduction in the shoot SOD activity of wheat, even at the lowest concentration of Cd.

**Fig. 5:**
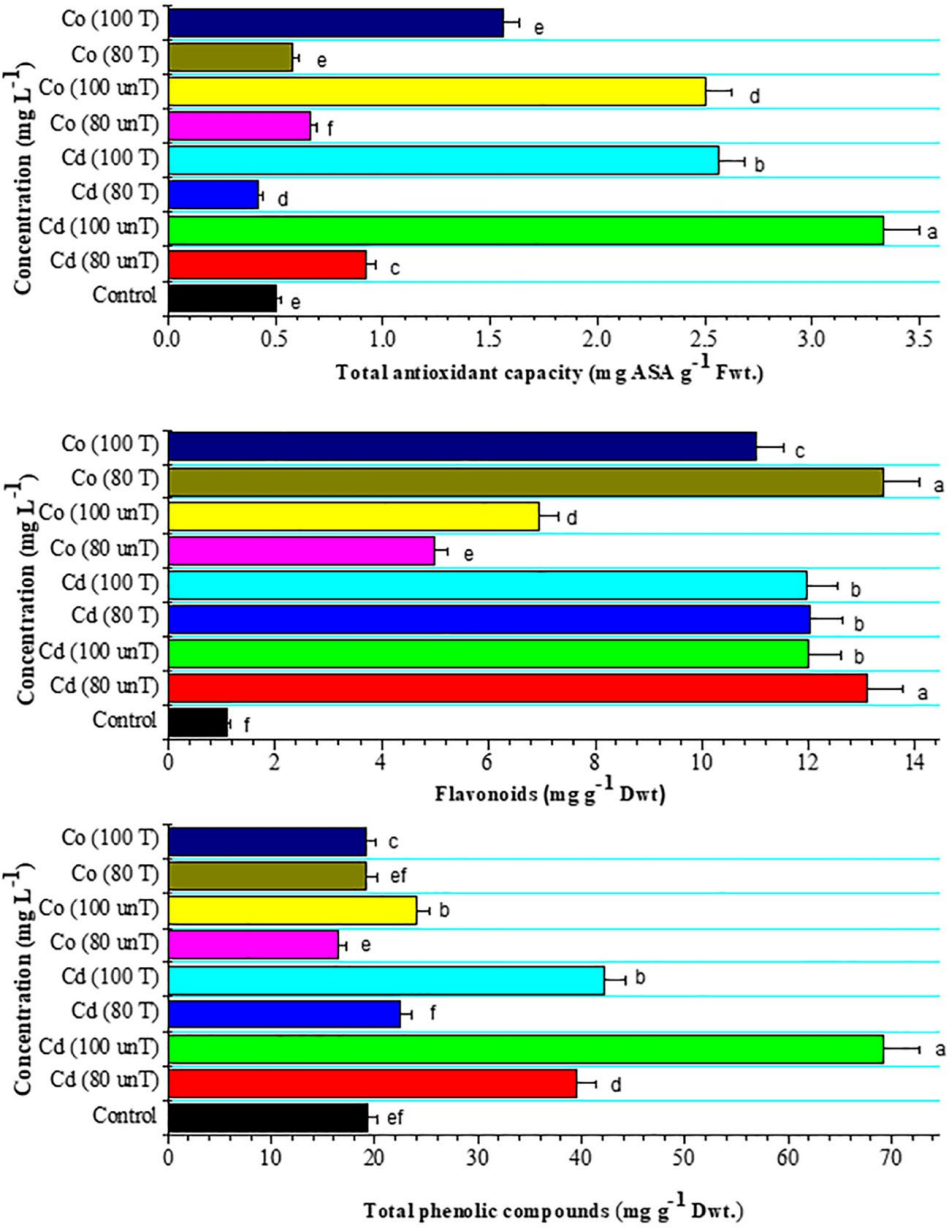
Effect of untreated and treated Cd and Co solutions (80 and 100 mg L^−1^) with *A. pinnata* (10%) on total phenolic compounds (mg g^−1^ D wt.), flavonoids (mg g^−1^ D wt), and total antioxidant capacity (mg ASA g^−1^ F wt.) activities of wheat seedlings after 21 days of cultivation. Means with different letters in each column are significantly different at *P* < 0.05. Standard error bars are shown above bars

Phenolic compounds have been described as electron-giving agents and antioxidants, acting as reducing agents, hydrogen donors, and singlet oxygen quenchers. They prevent the evolution of oxidant-free radicals and reactive species derived from metal catalysis (Kumar et al. 2020). Moreover, phenolics have the potential to function as biomarkers of metal exposure (Białońska et al. 2007). High Cd concentration increased the leaf content of phenolics, flavonoids, and total antioxidant capacity. In the present study, the levels of phenolics and flavonoids were higher at 100 mg L^−1^ metal concentration compared to 80 mg L^−1^ (Fig. 5). The increase in total phenolics in stressed plants was associated with an increase in flavonoid content (Fig. 5). This result agreed with the previous study results of Kısa et al. (2016). They found that Cd stress enhances the total phenolic compounds in maize leaves as compared to the control. In the present study, total phenolic compounds decreased at 80 mg L^−1^ of Co concentration, while they increased at 100 mg L^−1^ compared to the control (Fig. 5). Because of their high number of hydroxyl groups, chlorogenic, caffeic, and ferulic acids are among the most effective antioxidant phenolics (Marchiosi et al. 2020). Accordingly, they can sequester metals, acting as chelators. Wagay et al. (2020) reported that phenolics play a critical role in increasing the heavy metal tolerance in plants. It was found that CdCl_2_ reduced the number of free phenolics such as chlorogenic acid, ferulic acid, caffeic acid, and vanillic acid in the leaves of *Lepidium sativum* (Elguera et al. 2013). This effect may be due to the production of phenoxyl radicals by the antioxidative process (Sakihama et al. 2002). Another theory for the decrease in phenolic compounds in plants is that Cd overload has hampered the antioxidative system responses based on phenolics and other compounds to the point where plants are unable to produce new phenols (Tuladhar et al. 2021).

In the present study, flavonoids were significantly increased by the application of Cd and Co at 80 and 100 mg L^−1^ concentrations (Fig. 5). Flavonoids are produced to efficiently balance the produced oxidative destruction under stress. Flavonoids may achieve their antioxidant abilities by blocking the generation of ROS through their ability to chelate transition metal ions such as Fe and Cu (Nobahar et al. 2021).

In this study, the overall antioxidant capacity of the wheat leaf is measured and represented in Fig. 5. The total antioxidant capacity was significantly increased by increasing the Cd concentration to 80 and 100 mg L^−1^ treatment to become 0.92 and 3.33 mg ASA g^−1^ F wt, respectively, while the antioxidant capacity (2.50 mg ASA g^−1^ F wt) increased only at 100 mg L^−1^ of Co application. Many authors have shown a link between tolerance to heavy metal stress and an effective antioxidative defense mechanism (e.g., AbdElgawad et al. 2020; Hoque et al. 2021). Plant-metal interactions have similar mechanisms to other plant-abiotic conditions, and responses involve defensive non-enzymatic antioxidant systems. The present results showed that the increment of total antioxidant capacity was correlated with total phenolic content. This suggested the role of phenolics as scavengers of free radicals acting in conjunction with SOD, CAT, and POX to balance the increase of ROS levels. Similar findings were reported by Contreras et al. (2018).

According to the results in Figs. 3, 4, and 5, the use of *A. pinnata* as a metal phytoremediator of the original solutions of Cd and Co improves the enzymatic and non-enzymatic antioxidant status of the wheat seedlings. The use of treated solutions of Cd at 80 and 100 mg L^−1^ with *A. pinnata* reduces the production of ROS H_2_O_2_, lipid peroxides, and proline content. Moreover, the antioxidant enzyme activities were retained at their original activity in control (Fig. 4). Total phenolic and flavonoid compounds were decreased in treated wheat with treated solutions. The ability of *A. pinnata* to bio-filtrate toxic metals was demonstrated by many investigators. This result reflects the ability of *A. pinnata* to purify the metal-polluted solution to be more usable. The uptake of metals by the biomass of *A. pinnata* usually occurs through biosorption and insertion of the metal inside the cells (Saralegui et al. 2021).

## Conclusion

In this study, *A. pinnata* has been found to have the potential to mitigate the repressive effects of Cd and Co (at 80 and 100 mg L^−1^) polluted water on the germination and seedling growth of wheat (*Triticum aestivum* L.). The results revealed that the maximum removal efficiency (RE) was obtained in the control treatment on the fifth day, especially for Cd. Untreated Cd and Co solutions reduced germination, plumule and radicle lengths, amylase activity, and soluble sugar content of wheat seeds while increasing radicle phytotoxicity. The inclusion of *A. pinnata* in the germination media increased all the measured variables and decreased the radicle phytotoxicity by > 50% compared to the untreated solution. Cadmium at 80 and 100 mg L^−1^ decreased the fresh and dry biomass, as well as the height, of wheat seedlings after 21 days of culture when compared to Co. Cd and high Co concentrations increased the levels of H_2_O_2_, proline, MDA, phenolic, and flavonoid compounds. The activity of defensive antioxidant enzymes such as catalase and peroxidase was increased; however, superoxide dismutase activity was enhanced at 100 mg L^−1^ Cd and Co. In comparison to the control, the application of treated Cd and Co solutions by *A. pinnata* resulted in a decline in H_2_O_2_, proline, phenolic, and flavonoid component levels, as well as a reduction in catalase and peroxidase. Accordingly, the dry biomass of *A. pinnata* is an efficient phytoremediator for the heavy metals from irrigation water.

## Supplementary Information

Below is the link to the electronic supplementary material.

Supplementary file1 (DOCX 15 KB)

## Data Availability

Not applicable.
