# Supplementary material for: Alleviating the toxic effects of Cd and Co on the seed germination and seedling biochemistry of wheat (Triticum aestivum L.) using Azolla pinnata
Source: Environ Sci Pollut Res Int. 2023 May 26;30(30):76192–203. doi: 10.1007/s11356-023-27566-1 (PMC10293394; doi:10.1007/s11356-023-27566-1)
Supplement: Supplementary file 1 — Supplementary file1 (DOCX 15 KB) [file 11356_2023_27566_MOESM1_ESM.docx]

**Table S1.** Physico-chemical analysis of Peat moss

| **Parameters** | **Peat moss analysis** |
| --- | --- |
| Intermediate particles | 0.97 to 2 mm |
| Fine particles | < 0.3 mm |
| Bulk density | 0.97 g cm^3^ |
| Specific gravity | (Gs): 1.52 |
| Total porosity | 71 - 95.1% |
| Pore diameter | 0.1 – 4.6 mm |
| pH | 3.8 |
| EC (ds m^-1^) | 0.2 |
| Total Organic Carbon (g Kg^-1^) | 504.5 |
| Organic matter (g Kg^-1^) | 968.6 |
| Total Nitrogen (g Kg^-1^) | 9.5 |
| Total phosphorous (g Kg^-1^) | 0.2 |
| Total Potassium (g Kg^-1^) | 0.3 |
| C / N ratio | 52.9 |
| Ca (g Kg^-1^) | 2.8 |
| Mg (g Kg^-1^) | 1.1 |
| Na (g Kg^-1^) | 0.4 |
| Fe (g Kg^-1^) | 0.7 |
| Zn (mg Kg^-1^) | 13.5 |
| Cu (mg Kg^-1^) | 5.2 |
| Mn (mg Kg^-1^) | 16.0 |
| Cr (mg Kg^-1^) | 2.3 |
| Ni (mg Kg^-1^) | 4.5 |
| Cd (mg Kg^-1^) | 0.1 |
| Pb (mg Kg^-1^) | 5.1 |
